# Effects of jet nebulization on ventilator performance with different invasive ventilation modes: A bench study

**DOI:** 10.3389/fmed.2022.1004551

**Published:** 2022-10-19

**Authors:** Xinyu Li, Wei Tan, Hongwen Zhao, Wei Wang, Bing Dai, Haijia Hou

**Affiliations:** Department of Respiratory and Critical Care Medicine, The First Hospital of China Medical University, Shenyang, China

**Keywords:** jet nebulizer, mechanical ventilation, ventilation mode, ventilator performance, ventilator parameter

## Abstract

**Background:**

The effects of jet nebulization on ventilator performance in the volume control mode (VC) and pressure control mode (PC) of ventilation have not been determined.

**Objectives:**

The present study investigated the impact of jet nebulization on ventilator performance in different modes *in vitro*.

**Methods:**

Two types of jet nebulizer (ventilator-integrated jet nebulizers, external jet nebulizer) and six types of ventilator were connected with a simulated lung to simulate aerosol therapy during mechanical ventilation. The ventilation modes were set to VC and PC, and the driving flows of external jet nebulizer were set at 4 L/min and 8 L/min, respectively. Jet nebulizers were placed between patient airway and Y-piece or at 15 cm from the Y-piece in the inspiratory limb. The effects of jet nebulization were compared with the baseline of triggering performance, control performance, and tidal volume under different experimental conditions.

**Results:**

Ventilator-integrated jet nebulizers had no effect on ventilator performance in different modes (all *P* > 0.05). However, the effects of external jet nebulizers on ventilator performance varied widely: for triggering performance, all parameters were increased in different modes and nebulization positions (all *P* < 0.05), including the time from the beginning of the inspiratory effort to the lowest value of airway pressure needed to trigger the ventilator (TP_min_), the time to trigger (T_trig_), and the magnitude of airway pressure drop needed to trigger (P_trig_); for control performance, peak inspiratory pressure (P_peak_) and peak inspiratory flow(P_flow_) were increased in the VC mode (*P* < 0.05), but not significantly changed in the PC mode (*P* > 0.05);the actual tidal volume (VT) and expiratory tidal volume monitored (VTe) were significantly increased (*P* < 0.05), however, the inspiratory tidal volume monitored (VTi) was not affected by jet nebulization in the VC mode. In the PC mode, there were no significant changes in VT, whereas VTi decreased and VTe increased (*P* < 0.05). The higher the driving flow of external jet nebulizers, the stronger the impact on ventilator performance (all *P* < 0.05).

**Conclusion:**

Triggering performance was decreased in both the VC and PC modes when using an external jet nebulizer, while the effects of nebulization on control performance and tidal volume varied significantly.

## Introduction

Clinically, most patients who receive mechanical ventilation (MV) also require aerosol inhalation therapy to deliver bronchodilators, corticosteroids, antibiotics, and mucolytics (1–3). Jet nebulizers, ultrasonic nebulizers, and vibrating mesh nebulizers are commonly used for aerosol therapy during MV (4). Jet nebulizers are still among the most extensively used tools applied in patients received MV because they are readily available, cost-effective, and simple to manipulate (5, 6). A few ventilators are equipped with ventilator-integrated jet nebulizers, and the driving flow is provided by a branch of the inspiratory phase air flow of the ventilator, which will not affect ventilator performance (6, 7). However, a large proportion of ventilators are equipped with external jet nebulizers, and the driving flow is provided by external expressed air or an oxygen source (8).

Previous study has demonstrated the benefit of volume-controlled ventilation to increase aerosol delivery in comparison with a spontaneous breathing pattern in pressure support (9). Another two studies that have featured a simulated lung driven by an external ventilator or manual method, researchers have observed that nebulizers with external driving flow may lead to ineffective triggering (10, 11). The detailed effects of driving flow on patient-ventilator synchrony remains unclear. Mercier et al. (12) found that external jet nebulization had differential effects on tidal volume for different ventilation modes (volume control mode, pressure control mode) in a child model. Wang et al. (13) found that neither inspiratory tidal volume (VTi) nor expiratory tidal volume (VTe) monitored by ventilator accurately reflected the patient's actual tidal volume (VT) in either the volume control mode (VC) or pressure control mode (PC) mode. However, they only tested one ventilator in simulated lung, and a visual determination of VT was roughly recorded through the rise and fall of the water level. Effects of jet nebulization on ventilator performance with different invasive ventilation modes remained to be further investigated.

The studies mentioned above (10–13) were conducted over a long period and had certain limitations, such as antiquated ventilators and relatively simple evaluation indicators, measurement methods, and experimental conditions. We speculated that the effects of jet nebulization on ventilator performance may be extremely complex due to potential interactive factors such as ventilation modes, nebulization positions, the rate of driving flow, and the models of ventilators. Therefore, here, we comprehensively investigated the effects of jet nebulization on ventilator performance (including triggering performance, control performance, and VT) among six models of ICU ventilator (three of which were equipped with nebulization functions) with different invasive ventilation modes, driving flows, and nebulization positions using an active servo-simulated lung model.

## Materials and methods

### Simulated lung model setting

An active servo-simulated lung (ASL 5,000; IngMar, USA) is an intelligent respiratory simulation system that can precisely simulate the various respiratory mechanics of different lung diseases with preset parameters. Users can set the corresponding compliance, resistance, and inspiratory negative pressure generated by an inspiratory muscle according to different models of lung disease. The parameters used in this study to simulate a COPD patient were adapted from previous publications (14–16), as follows: compliance 60 mL/cmH_2_O, inspiratory resistance 10 cmH_2_O/L/s, expiratory resistance 15 cmH_2_O/L/s, and maximum drop in inspiratory pressure −5 cmH_2_O. To simulate the negative pressure produced by the respiratory muscles, 5% of the respiratory cycle time was set to active inspiration, 3% was set to end-inspiratory hold, and 15% was set to return pressure to the baseline. The respiratory rate was set at 15 breaths/min.

### Ventilator setting

Six models of ICU ventilator were used: E360 (Newport, Costa Mesa, USA), Servo-s (MAQUET, Karlsruhe, Germany), PB840 (Puritan Bennett, Missouri,USA), Vela (bird, Delaware, USA), Raphael (Hamilton, Bonaduz, Switzerland), and Evita-4 (Drager, Schleswig-Holstein, Germany) which were easily accessible in our intensive care unit and without any conflicts of interest. Among these, three models (Vela, Raphael, and Evita-4) were integrated with jet nebulization. All tested ventilators were set to flow-trigger mode, and trigger sensitivity was set to the most sensitive level to avoid auto-triggering or ineffective triggering. The fraction of inspiration oxygen (FiO_2_) was 21%, and the positive end expiratory pressure (PEEP) was 4 cmH_2_O. The backup respiratory rate was set at 10 breaths/min. The ventilation modes were set to VC and PC, respectively. In the VC mode, the preset tidal volume was 500 mL and the peak flow was 40 L/min with deceleration waveform, while in PC mode, the inspiratory pressure was 15 cmH_2_O above PEEP, the pressure rise slope was 20%, and the inspiratory time was 0.9 s.

### Simulate aerosol inhalation therapy during mechanical ventilation

**Figure 1:**
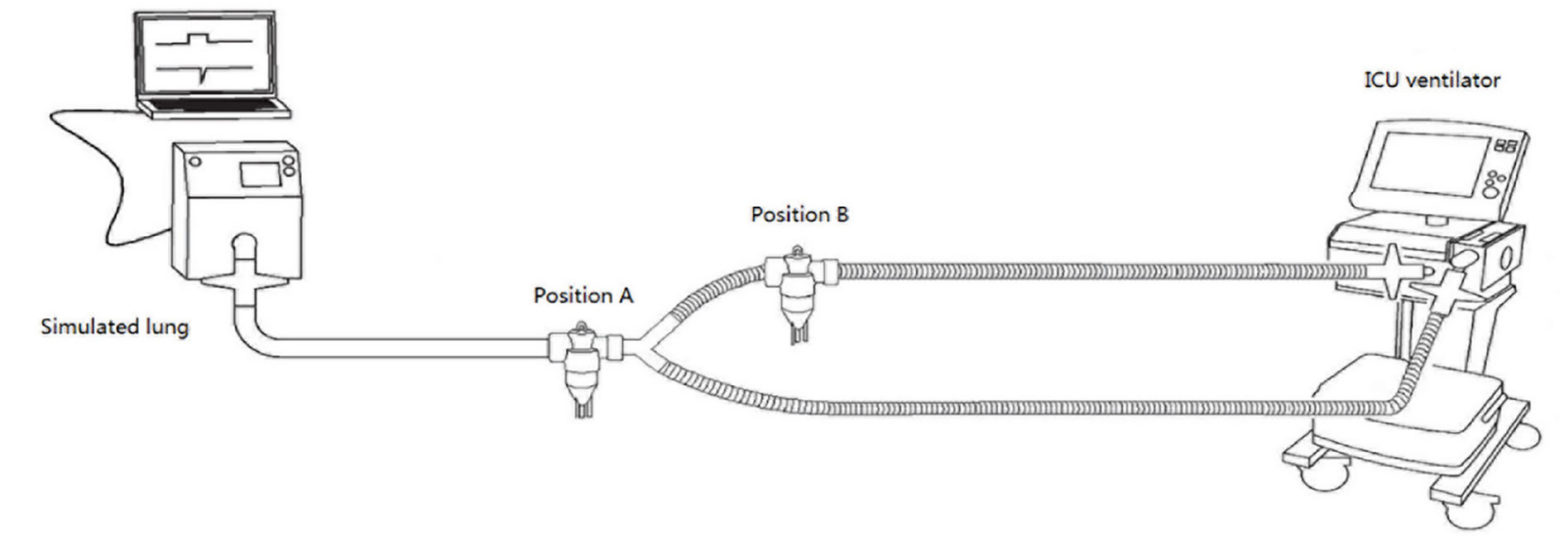
Diagrammatic illustration of the experiment.

All ventilators were connected to the simulated lung with a standard double-limb breathing circuit (RT100, Fisher & Paykel, Auckland, New Zealand) without a humidifier. The ventilator-integrated jet nebulizer and the external jet nebulizer (SVN 1884; Teleflex, Mexico) were filled with 3 mL purified water. The ventilator-integrated jet nebulizer was connected to the ventilator with a flexible tubing, while the external jet nebulizer was connected to a wall oxygen source (50 psi) through an oxygen meter (The Pacific Medical, Taiwan), and the driving flow was set to 4 and 8 L/min, respectively. Jet nebulizers were placed between patient airway and Y-piece (Position A) and at a 15 cm distance from the Y-piece in the inspiratory limb (Position B), as shown in Figure 1.

### Data collection

**Figure 2:**
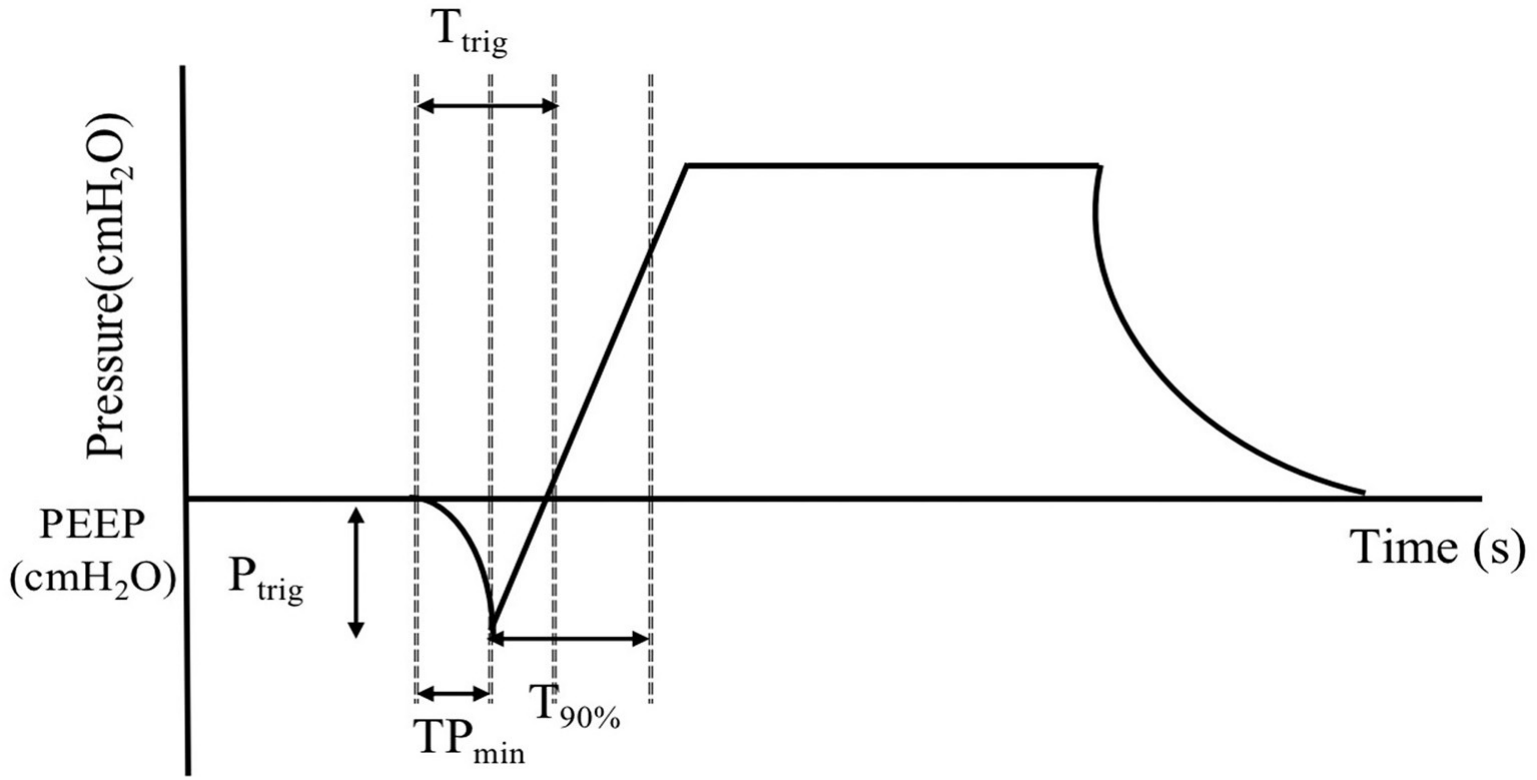
Graphic explanation of the variables. Time from the beginning of inspiratory effort to the lowest value of airway pressure needed to trigger the ventilator (TP_min_). Time to trigger (T_trig_). The magnitude of airway pressure drop needed to trigger (P_trig_). Time from ventilator triggering until airway pressure achieves 90% of the maximal pressure level during inspiration (T90%). Positive end expiratory pressure (PEEP).

Baseline data were collected before nebulization. Then, 3 min after nebulization, 10 consecutive breathing cycles of the following parameters were recorded, using the in-built software of the simulated lung. Three parameters were used to represent trigger performance: TP_min_ (time from the beginning of the lung simulator's inspiratory effort to the lowest value of airway pressure needed to trigger the ventilator), T_trig_ (time to trigger), and P_trig_ (magnitude of airway pressure drop needed to trigger). The next three parameters indicated control performance: peak inspiratory pressure (P_peak_), peak inspiratory flow (P_flow_), and time from ventilator triggering until airway pressure achieved 90% of the maximal pressure level during inspiration (T90%). The other parameters were VT (tidal volume displayed on the simulated lung), VTi, and VTe, which were displayed on the ventilator. Frequency of auto-triggering and ineffective triggering were also recorded. Figure 2 presents a graphic explanation of some parameters.

### Statistical analysis

The effects of jet nebulization on ventilation were evaluated using the relative percentage value: Relative% = [(actual value–baseline)/baseline] × 100%. The paired *t*-test was used to compare observed parameters pre- or post-nebulization, and further evaluate the effects of different driving flows and nebulization positions. As reported in previous studies, the frequent occurrence of statistically significant differences can be attributed to the small standard deviations observed in a bench study (10, 12). Differences were considered significant only when they were both statistically significant (*P* < 0.05) and clinically important (>10%) (14, 17–19). Statistical analysis was performed with the statistical software package SPSS (version 26.0; SPSS; Chicago, IL, USA).

## Results

### Effects on triggering performance

**Figure 3:**
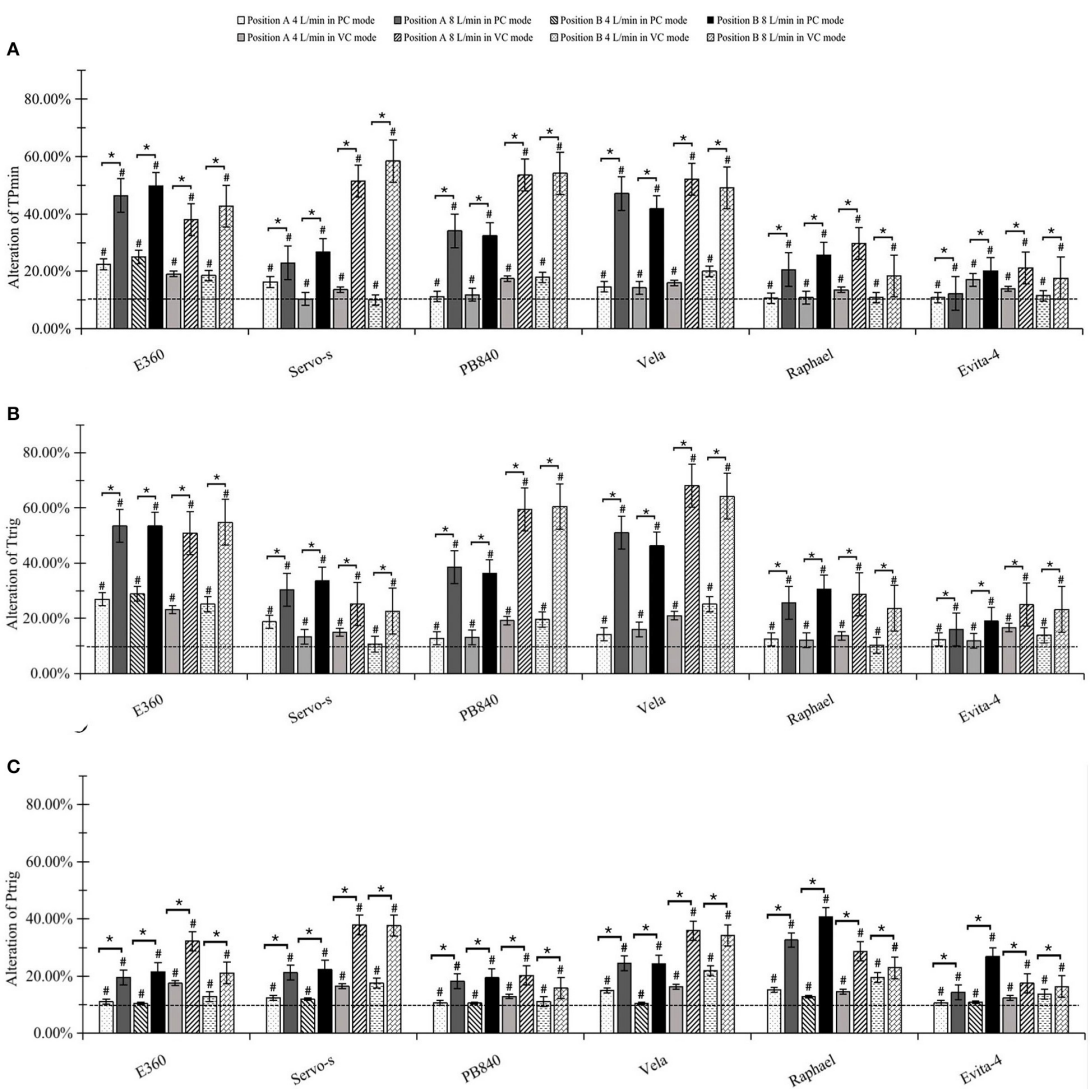
Effects on trigger performance. **(A)** Alteration of triggering response time (TP_min_). **(B)** Alteration of time to trigger (T_trig_). **(C)** Alteration of pressure to trigger (P_trig_). ^#^significant differences both statistically and clinically between pre-and post-nebulization. *significant differences between 4 and 8 L/min driving flow.

For both ventilator-integrated jet nebulizer and the external jet nebulizer, no auto-triggering or ineffective triggering was found during nebulization in any of the tested ventilators in the different modes and driving flows. For integrated jet nebulizers, TP_min_, T_trig_, and P_trig_ were not significantly altered by nebulization in any experimental condition. For external jet nebulizers, TP_min_, T_trig_, and P_trig_ were significantly increased after nebulization in different modes and nebulizer positions (all *p* < 0.05). As shown in Figure 3, the greater the driving flow, the stronger its impact on TP_min_, T_trig_, and P_trig_. The nebulization position, however, did not significantly affect triggering performance (*P* > 0.05).

### Effects on control performance

**Figure 4:**
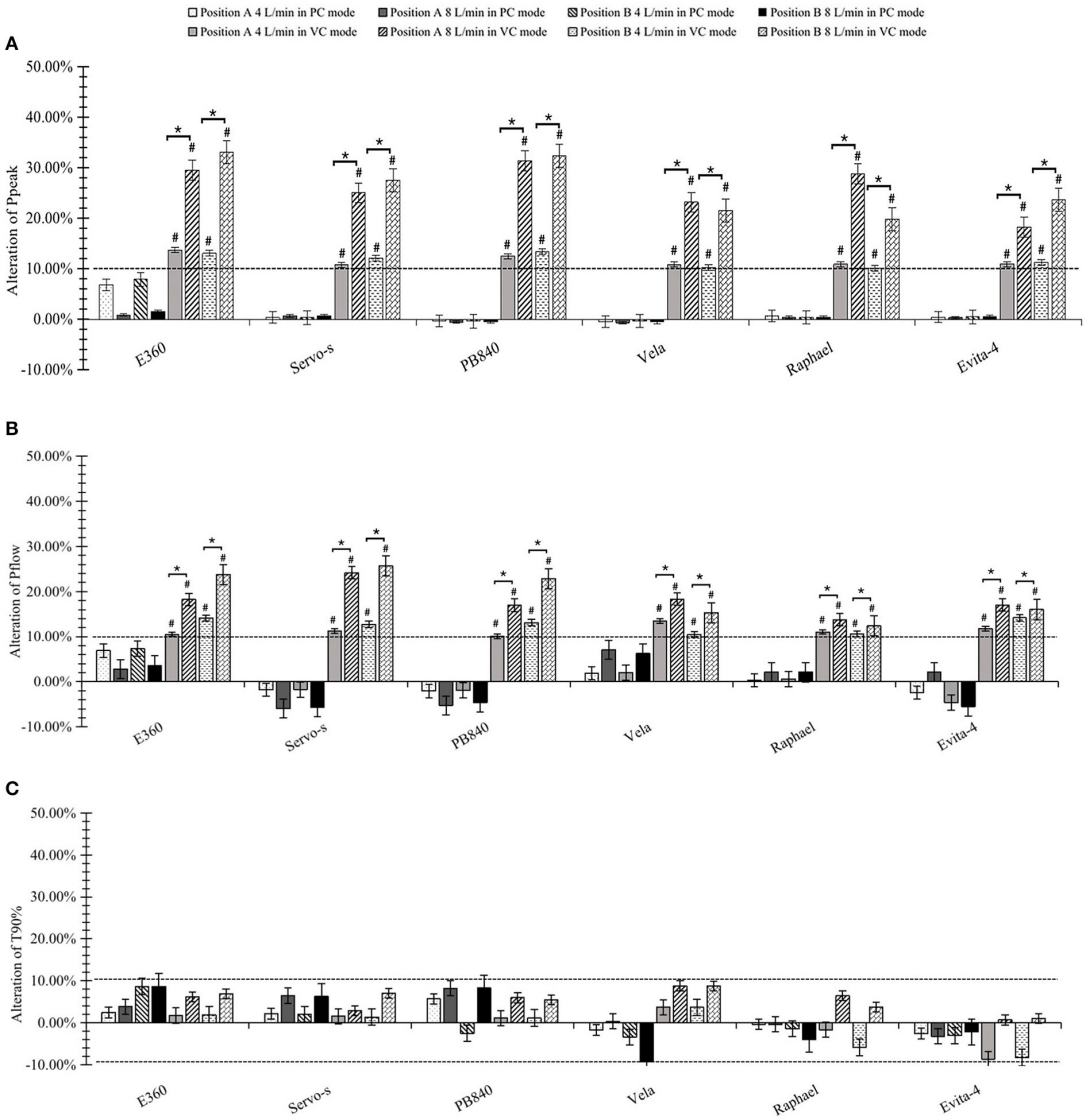
Effects on control performance. **(A)** Alteration of peak inspiratory pressure (P_peak_). **(B)** Alteration of peak inspiratory flow (P_flow_). **(C)** Alteration of time from ventilator triggering until airway pressure achieved 90% of the maximal pressure level during inspiration (T90%). ^#^significant differences both statistically and clinically pre- or post-nebulization. *significant differences between 4 and 8 L/min driving flow.

There were no significant differences among P_peak_, P_flow_, and T90% when using integrated jet nebulizers. As shown in Figure 4, external jet nebulizers had a significant effect on P_peak_ and P_flow_ in the VC mode (*p* < 0.05). The greater the driving flow, the stronger the impact on P_peak_ and P_flow._ However, external jet nebulizers did not have a significant effect on P_peak_, P_flow_, or T90% in the PC mode (all *p* > 0.05). Nebulization position also did not significantly affect them (*p* > 0.05).

### Effects on tidal volume

**Figure 5:**
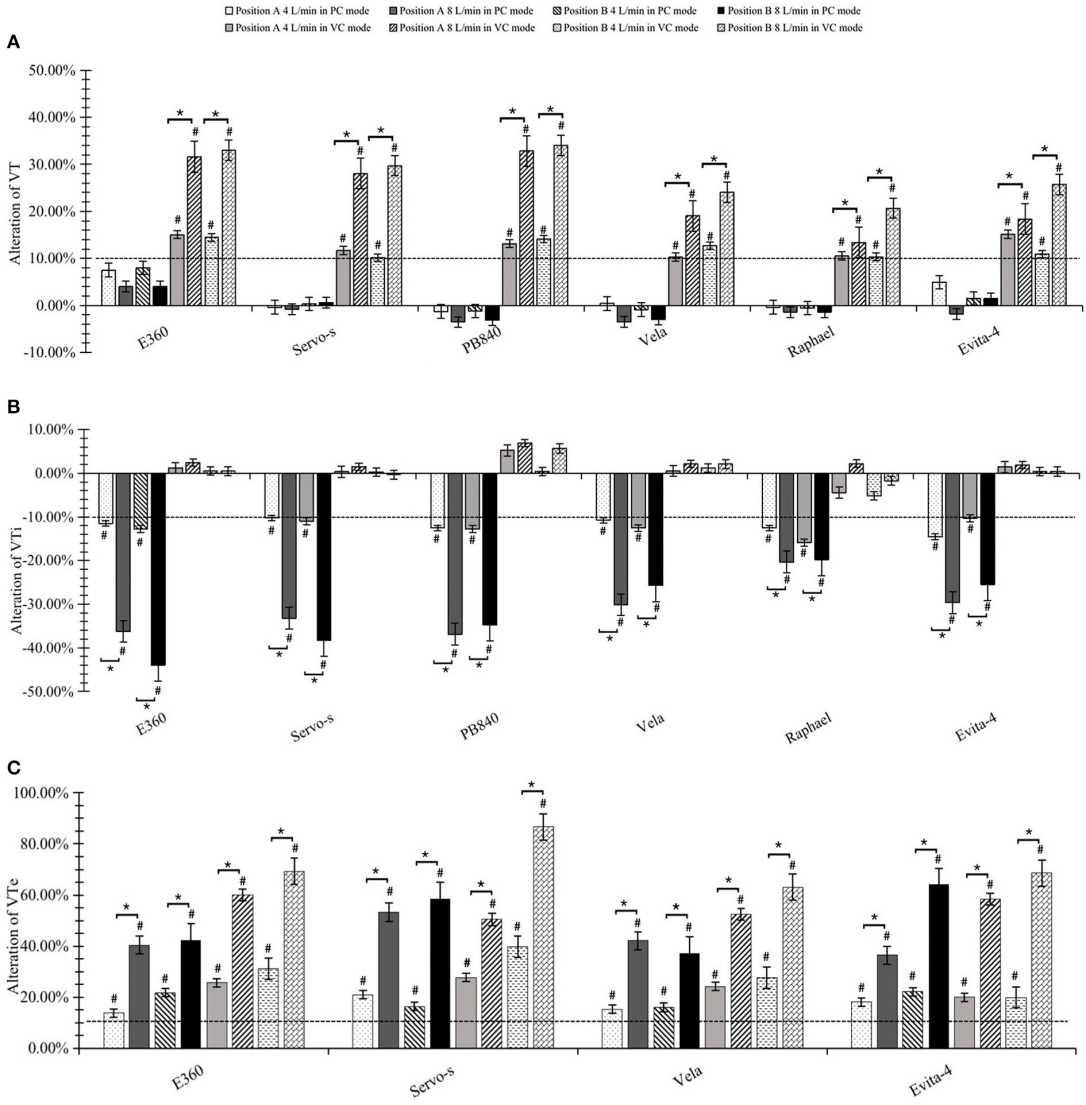
Effects on tidal volume. **(A)** Alteration of actual tidal volume. **(B)** Alteration of inspiratory tidal volume (VTi). **(C)** Alteration of expiratory tidal volume (VTe). ^#^significant differences both statistically and clinically pre- or post-nebulization. *significant differences between 4 and 8 L/min driving flow.

The actual measures of tidal volume, VTi and VTe were not significantly affected by integrated jet nebulizers under any experimental condition (all *p* > 0.05). When using external jet nebulizers in VC mode, VT and VTe were significantly increased after nebulization (*p* < 0.05). The differences grew as the driving airflow increased. However, VTi seemed to be unaffected in the VC mode. In the PC mode, however, VT was not significantly affected by jet nebulization in any experimental condition, and greater driving flow resulted in a decrease in VTi and an increase in VTe (*p* < 0.05) (Figure 5).

## Discussion

This bench study comprehensively assessed the effects of jet nebulization on ventilator performance, administered with six ventilators (three of which were equipped with ventilator-integrated jet nebulizers) in different modes, nebulization positions, and driving flows. Ventilator-integrated jet nebulizers had no effect on ventilator performance. Triggering performance was significantly decreased in both the VC and PC mode when using external jet nebulizers, while the effects of nebulization on the control performance and tidal volume varied significantly across the modes. In the VC mode, P_peak_, P_flow_, VT, and VTe were significantly increased, while VTi was not altered. VTi was decreased and VTe was increased in the PC mode, but there was no effect on VT. The greater the driving flow, the stronger the impact on mechanical ventilation, which was not affected by nebulization positions.

In general, jet nebulizers, ultrasonic nebulizers, and vibrating mesh nebulizers are three commonly used nebulizers in mechanically ventilated patients during aerosol therapy. Neither ultrasonic nebulizers nor vibrating mesh nebulizers produce additional airflow, and they see limited use due to heating drugs and their high price. Jet nebulizers are among the most extensively used nebulization devices for their cost-effectiveness, simple operation, and reduction of nosocomial infection (5, 6, 20). A few ventilators equipped with built-in jet nebulization ports provide the nebulizer's driving flow in parallel with the gas delivered through the inspiratory limb and have no effect on tidal volume or peak pressure (21); our results support those findings. However, most ventilators need additional compressed gas for aerosol delivery. Previous studies have found that in the VC mode, additional airflow is liable to increase VT, which results in patient–ventilator asynchrony during mechanical ventilation, while VT and peak pressure are not altered in the PC mode (6, 7, 12, 13). However, in those studies, quite a few variables were assessed.

Currently, there are two manners of trigger used in ventilators, namely, flow triggering and pressure triggering. The former is more commonly used due to less delay and lower inspiratory effort (22). When flow triggering is activated, the ventilator also provides a baseline or bias flow during the expiratory phase. At the patient side, the flow is equal to the baseline flow minus the flow on the expiratory side. When patient-end flow achieves the preset trigger threshold, the ventilator delivers an inspiratory flow. Extra flow during jet nebulization tends to increase the baseline flow, resulting in the degradation of triggering performance. Previous studies have speculated that jet nebulization may lead to a decline in triggering performance, with neither false triggering nor auto-triggering observed (6, 7); thus, the effects of jet nebulization on triggering performance were not determined. In our study, we found that jet nebulization could contribute to the degradation of triggering performance (TP_min_, T_trig_, and P_trig_). During clinical practice, physicians should observe the patients closely to see if there is hypoventilation or patient-ventilator desynchronization due to poor triggering performance. If so, it is advisable to alter the trigger sensitivity, ventilator modes or revising the preset respiratory rate upwards to maintain sufficient ventilation. Although we did not investigate the manner of the pressure triggering, the increase in actual triggering pressure indirectly confirmed the decline of triggering performance during jet nebulization. We also found that the effect on triggering performance was noticeable as driving flow increased.

In the VC mode, the variables for tidal volume, flow, and respiratory rate are independently set and are controlled by the ventilator (23). The preset tidal volume, VT, VTi, and VTe should be equal when the dual-limb circuit is entirely closed (17). The volume ventilator is controlled by the flow transducer on the inspiratory side, which is only affected by the preset tidal volume. The monitored VTi ventilator is always equivalent to the preset tidal volume. Therefore, jet nebulization with external flow has no significant effect on VTi, as in our findings. In the closed dual-limb circuit, the extra flow during jet nebulization tends to increase the gas volume delivered to the patient, which augments the P_peak_, P_flow_, and VT. This inevitably increases the air flow at the expiratory side of the ventilator, that is, the VTe ventilator accelerates. However, in clinical practice, we should evaluate the influence of jet nebulization on VTs according to the VTe monitored at the expiratory side and then lower the preset tidal volume to avoid hyperventilation or appropriately regulate the alarm range of pressure and tidal volume since VT cannot be measured directly.

In the PC mode, the preset inspiratory pressure and inspiratory time dictate the air flow that the ventilator delivers. VT largely depends on the patient's inspiratory effort, airway resistance, and lung compliance (24). As a result, P_peak_, P_flow_, and VTs are not altered, in spite of external flow. Unlike the case of air flow in the respiratory circuit, driving flow during jet nebulization is lower but has a higher pressure, which may cause a transient decrease in the pressure and flow delivered by the ventilator through a negative feedback mechanism. Therefore, although there has been no significant change in the measures of P_peak_ and P_flow_ at the simulated lung side (equivalent to the patient end), the VTi decreases. Unfortunately, the pressure and flow in the inspiratory circuit have not been monitored. In addition, the assumption that whether the negative feedback mechanism causes the decline of VTi requires further study. In clinical practice, when jet nebulization is performed with external flow, the preset pressure or alarm range of the ventilator may not need to be regulated in the PC mode, but close attention should be paid to patients in case of patient–ventilator asynchrony in both the VC and PC modes.

Within the operating limits, with higher pressure or greater gas flow to the nebulizer, smaller aerosol particles are generated, along with more aerosol output and shorter nebulization times (25). The driving flow of various nebulizers ranges from 2 L/min to 10 L/min (26). In our study, the driving flow was set at 4–8 L/min, as recommend by the manufacturer. Consistent with previous studies, we found that the greater the driving airflow, the greater the effects of jet nebulization on ventilator performance (13). In other words, if a driving flow is chosen or set that is at the lower limit of the recommended range, the influence of the jet nebulizer on ventilator performance necessarily falls. However, whether this has a significant effect on aerosol delivery remains to be seen. Our study showed that ventilator-integrated jet nebulizers had no significant effects on ventilator performance in either the VC or PC mode. It should be noted that most ventilator-integrated jet nebulizers provide lower driving pressure (< 15 psi) than the compressed air pressure (50 psi) commonly used in hospitals. As the driving pressure decreases, the aerosol diameter increases, which may lower the aerosol delivery efficiency of jet nebulization (25).

This study had some limitations. First, the results were obtained from an *in vitro* study, so consistent experimental settings should be undertaken to ensure the repeatability of results. The findings need to be validated in further clinical research. Second, a limitation of the simulated lung (ASL 5,000) was that it could not simulate the patient's expiratory effort and cycle inspiration (25, 27), and thus the effect of nebulization on cycle performance remains to be investigated. Third, nebulization types, simulated lung models, ventilator types and parameters may potentially influence ventilator performance, which need to be explored in the future.

In conclusion, ventilator-integrated jet nebulizers had no significant effect on mechanical ventilation. However, when nebulization was performed with external jet nebulizers, triggering performance was decreased in both the VC and PC modes. In the VC mode, P_peak_, P_flow_, VT, and VTe were significantly increased, but there were no significant changes in VTi; in the PC mode, neither control performance (P_peak_, P_flow_, and T90%) nor VTs were affected by jet nebulization, whereas VTi decreased and VTe increased. The higher the driving flow of external jet nebulizers, the greater the effect on ventilator performance.

## Data availability statement

The original contributions presented in the study are included in the article/supplementary material, further inquiries can be directed to the corresponding author.

## Author contributions

XL and WT contributed statistical analysis, data interpretation, drafting of manuscript, and final approval of the manuscript. HZ and WW contributed study design and final approval of the manuscript. BD and HH contributed critical revision. All authors contributed to the article and approved the submitted version.

## Funding

The research was supported by the Science and Technology Planning Project of Shenyang (No. 21-172-9-12).

## Conflict of interest

The authors declare that the research was conducted in the absence of any commercial or financial relationships that could be construed as a potential conflict of interest.

## Publisher's note

All claims expressed in this article are solely those of the authors and do not necessarily represent those of their affiliated organizations, or those of the publisher, the editors and the reviewers. Any product that may be evaluated in this article, or claim that may be made by its manufacturer, is not guaranteed or endorsed by the publisher.
